# Targeting Xcr1 on Dendritic Cells Rapidly Induce Th1-Associated Immune Responses That Contribute to Protection Against Influenza Infection

**DOI:** 10.3389/fimmu.2022.752714

**Published:** 2022-02-28

**Authors:** Demo Yemane Tesfaye, Sonja Bobic, Anna Lysén, Peter Csaba Huszthy, Arnar Gudjonsson, Ranveig Braathen, Bjarne Bogen, Even Fossum

**Affiliations:** ^1^ Department of Immunology, Division of Laboratory Medicine, Oslo University Hospital, Oslo, Norway; ^2^ Kristian Gerhard Jebsen Center for Research on Influenza Vaccines, University of Oslo and Oslo University Hospital, Oslo, Norway; ^3^ Center for Immune Regulation, Institute of Immunology, University of Oslo and Oslo University Hospital Rikshospitalet, Oslo, Norway

**Keywords:** dendritic cells, XCR1, Th1, IgG2a, targeting

## Abstract

Targeting antigen to conventional dendritic cells (cDCs) can improve antigen-specific immune responses and additionally be used to influence the polarization of the immune responses. However, the mechanisms by which this is achieved are less clear. To improve our understanding, we here evaluate molecular and cellular requirements for CD4^+^ T cell and antibody polarization after immunization with Xcl1-fusion vaccines that specifically target cDC1s. Xcl1-fusion vaccines induced an IgG2a/IgG2b-dominated antibody response and rapid polarization of Th1 cells both *in vitro* and *in vivo*. For comparison, we included fliC-fusion vaccines that almost exclusively induced IgG1, despite inducing a more mixed polarization of T cells. Th1 polarization and IgG2a induction with Xcl1-fusion vaccines required IL-12 secretion but were nevertheless maintained in BATF3^-/-^ mice which lack IL-12-secreting migratory DCs. Interestingly, induction of IgG2a-dominated responses was highly dependent on the early kinetics of Th1 induction and was important for optimal protection in an influenza infection model. Early Th1 induction was dominant, since a combined Xcl1- and fliC-fusion vaccine induced IgG2a/IgG2b polarized antibody responses similar to Xcl1-fusion vaccines alone. In summary, our results demonstrate that targeting antigen to Xcr1^+^ cDC1s is an efficient strategy for enhancing IgG2a antibody responses through rapid Th1 induction, which can be utilized for improved vaccine design.

## Introduction

Conventional dendritic cells (cDCs) capture and process foreign antigens for presentation of antigen-specific T cells. Through this function, cDCs have a central role in the initiation phase of the cellular immune response and can as a consequence influence the polarization of the ensuing immune responses (1).

cDCs can be identified as MHC-II^+^CD11c^+^ cells and can be further divided into two subpopulations on the basis of functional and ontogenic differences (2). cDC1s are able to cross-present antigen to CD8^+^ T cells (3, 4) and selectively express the surface receptor Xcr1 (5, 6), while cDC2s can be identified based on express CD11b and SIRP1α expression (7, 8). Previous studies have indicated that cDC1s preferentially polarize the CD4^+^ T cell response toward Th1, while cDC2 polarize toward Th2 (9, 10), suggesting that targeting antigens toward specific DC subsets can be a valid strategy for influencing the polarization of the vaccine-induced immune responses. Antigens can be targeted directly to cDCs by fusion to antibodies, chemokines, or other ligands that bind surface receptors expressed on the cDCs [reviewed in (1, 11, 12)]. Such approaches have been shown to enhance antigen-specific immune responses in mice as well as in larger animals (13–17).

In this study, we deliver antigen to cDC1s by genetic fusion to the chemokine Xcl1, the ligand of the Xcr1 receptor. Xcl1-fusion vaccines have been demonstrated to enhance CD8^+^ T cell response (13, 14, 18) and to induce a preferential IgG2a/IgG2b antibody response, associated with Th1 polarization (13, 19). While Xcl1 has previously been identified as a Th1-associated chemokine (20), it is unclear if the chemokine directly influences Th1 polarization when used for targeting antigens to cDC1s. As a second targeting strategy, we included antigens fused to flagellin (fliC) from *Salmonella typhimurium* that has been reported to induce a Th2-polarized response (21, 22) and which we have previously seen to induce an IgG1-dominated antibody response (22). FliC acts as a ligand for TLR5, which has been reported to be expressed on cDC2s (23) but also on pDCs and a specific subset of CD8^+^Xcr1^-^ DCs in skin-draining lymph nodes (LN) (24, 25). In addition, fliC is a ligand for the intracellular NLRC4–NAIP5 inflammasome activating complex (26, 27).

Through a series of *in vitro* and *in vivo* experiments, we evaluate the molecular and cellular requirements for Xcl1- and fliC-fusion vaccines to influence antibody and CD4^+^ T cell polarization. The results demonstrate that Xcl1- and fliC-fusion vaccines both induce IFNγ-secreting CD4^+^ Th1 cells, although with different kinetics. Our observations indicate that the kinetics of T cell polarization play a crucial role in determining the polarization of antibody responses.

## Material and Methods

### Cell Lines, Virus, and Antibodies

Human embryonic kidney (HEK) 293E cells (from ATCC) were used for the expression of HA and ovalbumin (OVA) fusion proteins. The HEK293E cells were cultured in complete RPMI media. Complete RPMI medium contains RPMI 164 (Invitrogen, Waltham, MA) supplemented with 40 mg/ml gensumycin (Sanofi-Aventis Norge AS, Lysaker, Norway), 50 μM monothioglycerol (Sigma, St. Louis, MO, USA), 1 mM sodium pyruvate, and 0.1 mM non-essential amino acids (Lonza, Walkersville, MD, USA). For serum ELISAs, ALP-conjugated anti-mouse IgG (Fc-specific) from Sigma (St. Louis, MO, USA) and anti-mouse IgG1-bio (clone 10.9), anti-mouse IgG2a-bio (clone 8.3), and anti-mouse IgG2b-bio (clone R12-3) from BD Pharmingen (San Diego, CA, USA) were used. For flow cytometric analysis, anti-CD3e (145-2C11, Tonbo Biosciences, San Diego, CA, USA), anti-CD19 (1D3, Tonbo Biosciences), anti-CD49b (DX5, eBioscience, San Diego, CA, USA), anti-Ly6G (1A8, Tonbo Biosciences), CD45R/B220 (RA3-6B2, Tonbo Biosciences), anti-MHCII (M5/114.15.2, BioLegend, San Diego, CA, USA), anti-CD11c (N418, Tonbo Biosciences), anti-CD11b (M1/70, Tonbo Biosciences), anti-CD24 (M1/69, BioLegend), anti-CD8α (53-6.7, BioLegend), anti-CD4 (GK1.5, BioLegend), anti-DO11.10 (KJ1-26, BioLegend), anti-CD14 (rmC5-3), anti-IFNγ (XMG1.2), anti-T-bet (eBio4B10, eBioscience), anti-GATA3 (TWAJ, Invitrogen, Carlsbad, CA, USA), and anti-RORγt (AFKJS-9, eBioscience) and were used.

### Mice

All animal experiments were approved by the Norwegian Food Safety Authority (NFSA). BALB/c mice aged 6–8 weeks were purchased from Janvier, France. BATF3^-/-^ mice bred on a BALB/c background were purchased from The Jackson Laboratory (Stock No.: 013755) and bred in-house. Mice were euthanized if they lose 80% of their original weight after influenza virus challenge as a human endpoint according to the guidelines of NFSA.

### Generation and Purification of Targeted Vaccines

Construction of fusion vaccines that contain targeting, dimerization, and antigenic domains has been described before (28). The targeting units used in this study were the chemokine ligand Xcl1 specific for Xcr1, the TLR5 ligand fliC, or a scFV specific for the hapten 4-hydroxy-3-iodo-5-nitrophenylacetic acid (NIP) as negative control. As antigens, aa 18-541 of HA from influenza A/PR/8/34 or full-length ovalbumin (OVA) was used.

Purification of fusion vaccine proteins was done as described in Gudjonsson et al. (29) with some modifications. In brief, HEK293E cells were seeded in 5-layer tissue culture flasks (Falcon Multi-Flasks) and transfected using polyethylenimine (PEI, 1 mg/ml stock) at a ratio of 500 μg PEI to 250 μg DNA. The supernatant was harvested after 4–5 days and applied on a CaptureSelect FcXL Affinity Matrix column (Life Technologies, Carlsbad, CA, USA) connected to an ÄKTAprime plus (GE Healthcare, Chicago, IL, USA). Bound fusion vaccines were washed with PBS, eluted in 0.1 M glycin–HCl pH 2.7, and immediately dialyzed twice against PBS. Purified fusion vaccines were concentrated using 10-Kd cutoff Vivaspin columns (Sartorius Stedim Biotech, Göttingen, Germany), aliquoted, and stored at -80°C until use.

### Intradermal DNA Vaccination of Mice

BALB/c mice were anesthetized by intraperitoneal injection of 150 µl ZRF mixture containing 250 mg/ml Zoletil Forte (Virbac, Carros, France), 20 mg/ml Rompun (Bayer Animal Health), and 50 μg/ml fentanyl (Actavis, Parsippany-Troy Hills, NJ, USA). After shaving the lower back, 25 µl of DNA vaccine (0.5 µg/µl in 0.9% NaCl) was injected intradermally (i.d.) on the left and right flanks. Immediately after injection, the skin was electroporated using the Derma Vax (Cyto Pulse Sciences, Inc., Glen Burnie, MD, USA) system with two pulses of 450 V/cm × 2.5 µs and eight pulses of 110 V/cm × 8.1 ms.

### Isolation of CD4^+^ T Cells From Spleen

Splenocytes from BALB/c, BATF3^-/-^, and DO11.10 mice were prepared using the GentleMACS dissociator (Miltenyi Biotec, Bergisch Gladbach, Germany) according to the manufacturer’s protocol. Briefly, spleens were dissociated in GentleMACS C tubes in complete RPMI media. Erythrocytes were lysed by incubation with ACT buffer for 5 min on ice. Finally, cells were filtered through a 70-μm nylon cell strainer. CD4^+^ T cells from DO11.10 mice spleens were isolated using a CD4^+^ isolation kit (Miltenyi Biotec), according to the manufacturer’s protocol.

### *In Vitro* Generation of Bone Marrow-Derived DCs

Bone marrow cells were harvested by flushing tibiae and femur with medium. The cell suspension was filtered through a 70-μm nylon cell strainer, and 1 × 10^7^ single-cell suspension in 5 ml total volume was seeded in a 6-well plate. Flt3L (PeproTech, Rocky Hill, NJ, USA) (0.1 μg/ml) was added, and the cells were incubated for 9 days at 37°C, 5% CO_2_ (30). Semi-adherent cells were subsequently harvested and analyzed by flow cytometry after staining with anti-CD45/B220, anti-CD11c, anti-CD11b, and anti-CD24 for 20 min on ice.

### Serum ELISA

High binding 96-well ELISA plates (Coster) were coated with inactivated PR8 virus (Charles River Laboratories, Wilmington, MA, USA) (1:1,600 in PBS) overnight (ON) at 4°C and blocked with 1% w/v BSA in PBS with 0.02% w/v Na azide for 1 h at room temperature (RT). Blood samples were collected from the saphenous vein of mice and sera isolated by two successive centrifugations for 5 min at 13,000 rpm. Serum samples were titrated down 3-fold starting from 1:50 in ELISA buffer (0.1% w/v BSA, 0.2% Tween, and 0.02% w/v in PBS) into the coated 96-well plate and incubated ON at 4°C. Next, the plates were washed (3×) and 50 μl of 1 μg/ml biotinylated anti-mouse IgG1[a], IgG2a[a], or IgG2b diluted in ELISA buffer was added and incubated for 1.5 h at RT. After washing (3×), the plates were incubated with 1:3,000 diluted streptavidin-ALP (GE Healthcare (RPN1234V)) for 45 min at RT. The plates were then washed (3×) and developed using 100 µl/well of substrate buffer (1 mg/ml phosphate substrate (Sigma, P4744)). After 30 min, OD_405_ was measured on a Tecan Sunrise spectrophotometer. The cutoff value for the Ab titer was determined by calculating the mean OD (+ 3 SD) of sera from NaCl-vaccinated control groups. The reciprocal of the highest serum dilution of a sample giving more OD than the cutoff is reported. If an OD value of a sample did not exceed that of the cutoff value, the sample was given an endpoint titer of 1.

### IFNγ ELISPOT

A single-cell suspension from spleen was prepared as described above. To detect IFNγ and IL4 secreted by splenocytes, ELISpotPLUS for mouse IFNγ and IL4 kit with precoated anti-IFNγ and anti IL4 plates, respectively, was used in accordance with the manufacturer’s protocol (Mabtech AB, Nacka Strand, Sweden). In short, spleens were dissociated, treated with Tris-buffered ammonium chloride (ACT) lysis buffer, and filtered through a 70-μm nylon strainer to prepare single-cell suspensions. Cells were added to the plates at a concentration of 0.5 × 10^6^ and restimulated with the HA-derived peptide HNTNGVTAACSHEG (MHC-II, I-E^d^-restricted) or a negative control peptide at a concentration of 2 μg/ml for 18 h at 37°C 5% CO_2_. The plates were automatically counted and analyzed using a CTL ELISPOT reader (CTL Europe GmbH, Bonn, Germany). The values obtained from the negative control peptide wells were subtracted from the values obtained from stimulation with specific peptides for each sample.

### *In Vitro* Th Polarization

OVA-specific CD4^+^ T cells were isolated from DO11.10 TCR transgenic mice by harvesting spleens and generating single-cell suspensions as described for isolation CD4+ T cells from spleen. DO11.10 CD4^+^ T cells were then purified using a CD4 T cell isolation kit (Miltenyi Biotec), according to the manufacturer’s protocol. Purified DO11.10 cells were seeded at a concentration of 5 × 10^4^ cells in 48-well plates together with 2.5 × 10^5^ BM DCs and 0.5 μg αNIP-, Xcl1-, or fliC-OVA in RPMI with 10% FCS. The plates were incubated for 72 h at 37°C with 5% CO_2_ before cells were harvested and analyzed by flow cytometry after staining for anti-CD4, anti-DO11.10, anti-T-bet, ant-GATA3, and anti-RORγt. Data were acquired on a Fortessa (BD) flow cytometer and analyzed using FlowJo software (FlowJo, LLC, Ashland, OR, USA).

### *In Vitro* Proliferation on Sorted Bone Marrow-Derived DCs

Bone marrow-derived cDC1s and cDC2s were defined as CD45R^-^CD11c^+^CD11b^-^CD24^+^ and CD45R^-^CD11c^+^CD11b^+^CD24^-^ cells, respectively, and sorted on a BD FACSMelody (BD Biosciences, Franklin Lakes, NJ, USA). Post-sorting evaluation confirmed a purity >99% for two bone marrow-derived DC (BMDC) populations. OVA-specific CD4^+^ DO11.10 cells were isolated as described above and stained with 5 μM CellTrace CTV before incubation with sorted DCs at a ratio of (3:1) and 1 μg/ml Xcl1-OVA, fliC-OVA, or αNIP-OVA for 4 days. As a positive control, cells were incubated with 0.5 μg/ml of the OVA_323-339_ peptide. Proliferation of DO11.10 cells were determined by flow cytometry on an Attune NxT (Thermo Fisher Scientific, Waltham, MA, USA).

### *In Vivo* Th Polarization

OVA-specific DO11.10 cells were isolated as for *in vitro* Th polarization, and 1 × 10^6^ cells transferred to naïve mice one day before intradermal immunization with 25 μg DNA encoding αNIP-, Xcl1-, or fliC-OVA. Inguinal and axillary LNs were harvested on specified days after immunization and single-cell suspensions generated using GentleMACS dissociator (Miltenyi Biotec). In short, LNs were placed in C MACS tubes containing complete RPMI medium dissociated by running program B on the GentleMACS dissociator. After filtration, the single-cell suspensions from LNs were filtered through a 70-μm nylon cell strainer washed in PBS and analyzed by flow cytometry after staining for anti-CD19, anti-CD14, anti-CD3, anti-CD4, anti-DO11.10, anti-T-bet, ant-GATA3, and anti-RORγt. Data were acquired on a Fortessa (BD) flow cytometer and analyzed using FlowJo software (FlowJo, LLC).

### IL-12 Blocking

*In vitro* cocultures of BMDC and DO11.10+ CD4+ T cells in the presence of Xcl1, fliC-, or αNIP-Ova were treated with 10 μg/ml of anti-IL12 (AF-419-SP, R&D Systems, Minneapolis, MN, USA) or an unspecific isotype antibody control for 72 h. DO11.10^+^CD4^+^ T cells were then evaluated for the expression of transcription factors and supernatants harvested for cytokine ELISA as described above.

*In vivo:* mice were vaccinated as described in the intradermal DNA vaccination. For the early time-point blockade, 500 μg of anti-IL12 antibody (clone R2-9A5) (Bio X Cell, Lebanon, NH, USA) was injected i.p 1 and 2 days after vaccination, while for the later time inhibition the anti-IL12 antibody was injected on days 6 and 7. Controls at each time point received an unspecific isotype control antibody.

### *In Vivo* Cytotoxicity

Cytotoxicity was performed as previously described (31). In short, splenocytes from BALB/c mice were incubated with the HA-derived MHC-I restricted peptide (IYSTVASSL) or an unspecific control peptide at a concentration of 1 μg/ml in 5 × 10^7^ cells/ml for 1 h at 4°C. Peptide-loaded cells were stained with 1.25 μM (negative control) or 12.5 μM (IYSTVASSL) CellTrace™ Violet (Thermo Fisher) for 30 min at 37°C, before they were washed 2× in PBS, resuspended in PBS at a concentration of 5 × 10^7^ cells/ml, and mixed 1:1. A total of 1 × 10^7^ cells were injected *i.v.* into BALB/c or BATF3^-/-^ mice that had been DNA immunized with 25 μg Xcl1-HA, fliC-HA, or NaCl 9 days prior. After 18 h, spleens were harvested and the ratio of CTV^low^ to CTV^high^ determined by flow cytometry. Cytotoxicity was calculated as % specific lysis = [1 - (Avg NaCl ratio/experimental ratio)] × 100.

### Statistics

All statistical analyses were performed using the GraphPad Prism 8 software. Significant differences in antibody responses, cytokine ELISA, or T-cell responses were calculated using the parametric t-test or non-parametric t-test (Mann–Whitney) when comparing two treatment groups and one-way ANOVA with multiple-comparison correction when comparing >2 groups (*p < 0.05, **p < 0.01, ***p < 0.001). Differences in antibody responses over time and weight curves after infection were calculated using two-way ANOVA (*p < 0.05, **p < 0.01, ***p < 0.001). Differences in survival were calculated by Mantel–Cox (*p < 0.05, **p < 0.01, ***< 0.001). Data and error bars are presented as mean ± SEM.

## Results

### Xcl1-Fusion Vaccines Induce Rapid Th1 Responses After DNA Vaccination

**Figure 1:**
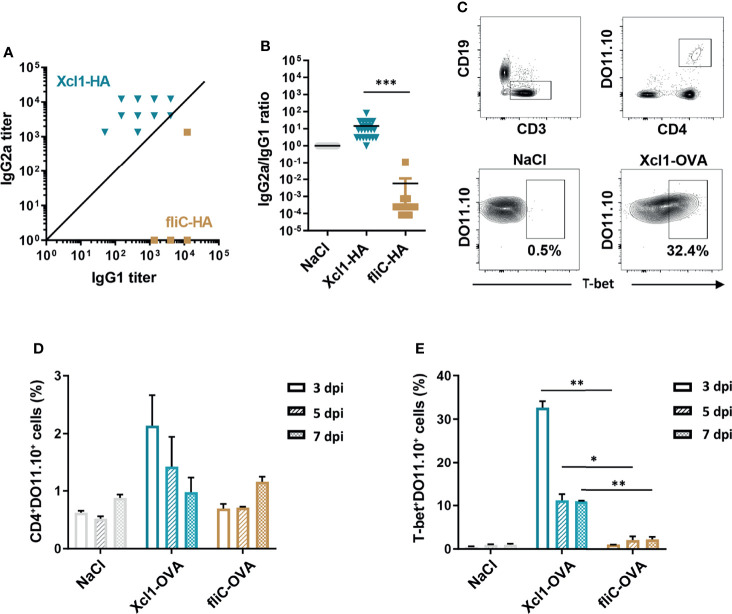
DNA immunization with Xcl1-HA induces rapid Th1-associated immune responses. **(A)** IgG1 and IgG2a anti-HA antibodies in sera from BALB/C mice obtained 2 weeks after a single i.d. DNA immunization/electroporation with 25 μg plasmid encoding Xcl1-HA or fliC-HA. **(B)** IgG2a/IgG1 ratios in single mice presented in **(A)**. **(C–E)** 1 × 10^6^ naïve DO11.10 cells were transferred to BALB/c mice that were subsequently immunized with 25 μg plasmid encoding Xcl1-OVA or fliC-OVA. Inguinal LNs were harvested 3, 5, or 7 days after vaccination. **(C)** Gating strategy for identification of CD4^+^DO11.10^+^ and expression of the transcription factor T-bet. **(D)** Percentage of CD4^+^DO11.10^+^ cells and **(E)** T-bet^+^ DO11.10 cells elicited by immunization. Data shown are either pooled from 2 independent experiments **(A, B)**, or representative of two independent experiments **(C–E)**, with 20 **(A, B)**, or 3 **(C–E)** mice per group. Statistical analysis was performed using the non-parametric t-test **(B)** or one-way ANOVA with Tukey’s multiple-comparison test comparing Xcl1-OVA and fliC-OVA for the different timepoints **(D, E)**. *p < 0.05, **p < 0.01, ***p < 0.001.

To better understand the mechanism of how targeting cDCs can influence the polarization of the immune response, we compared Xcl1- and fliC*-*fusion vaccine molecules, as these have previously been seen to induce differently polarized antibody responses (**Supplementary Figure 1A**) (28, 32). While Xcl1-fusion vaccines target the Xcr1 receptor which is specifically expressed on cDC1s (5, 6, 29), the surface receptor for fliC, TLR5, has been reported to be expressed on CD11b^+^ cDC2s (**Supplementary Figure 1B**) (23). However, when staining for TLR5 in spleen we only observed a small percentage of TLR5^+^ DC, although the percentage was higher on cDC2 than cDC1s (**Supplementary Figure 1C**). In addition, fliC has also been reported to activate the intracellular NLRC4–NAIP5 inflammasome activating complex, suggesting that fliC-fusion vaccines may enhance immune responses though several mechanisms. Intradermal (i.d.) DNA vaccination using Xcl1- or fliC-fusion vaccines containing hemagglutinin (HA) from influenza A/Puerto Rico/8/34 (PR8) as an antigen demonstrated that fliC-HA induced almost exclusively antibodies of the IgG1 subclass, while Xcl1-HA induced higher titers of IgG2a and IgG2b (**Figures 1A, B** and **Supplementary Figure 1D**) (22). Both vaccines did, however, induce protection against a lethal dose (50xLD_50_) of influenza A (PR8) (**Supplementary Figures 1E, F**).

The IgG subclass data suggest that Xcl1- and fliC-HA differentially influence Th polarization. To test this, IFNγ and IL4 ELISPOT assays were performed on splenocytes from BALB/C mice vaccinated by i.d. DNA immunization (**Supplementary Figures 2A, B**). Spleens were harvested 1 or 2 weeks after vaccination, and single-cell suspensions were stimulated with the MHC-II restricted HA peptide HNTNGVTAACSHEG. Already after 1 week, immunization with Xcl1-HA induced INFγ−secreting splenocytes (**Supplementary Figure 2A**). Neither of the vaccines induced IL4-secreting splenocytes above background at this time point (data not shown). Somewhat surprisingly, fliC-HA induced significantly higher numbers of IFNγ−secreting cells compared to Xcl1-HA immunized mice after 2 weeks, while there was no difference in IL4-secreting splenocytes (**Supplementary Figure 2B**).

The ELISPOT results suggest that Xcl1-fusion vaccines rapidly induce IFNγ−secreting splenocytes when delivered by i.d. DNA vaccination. To obtain a better understanding of the kinetics, we utilized DO11.10 transgenic mice that have CD4^+^ T cells with a TCR specific for the peptide OVA_323-339_ presented on the MHC-II molecule I-A^d^ (33). 1 × 10^6^ CD4^+^DO11.10 cells were injected i.v. into naïve BALB/c mice that were immunized 1 day later by i.d. delivery of DNA encoding Xcl1-OVA, fliC-OVA, or NaCl followed by electroporation. Draining LNs were harvested 3, 5, or 7 days after immunization and evaluated for proliferation and polarization of DO11.10 cells (**Figures 1C**–**E**). DNA immunization with Xcl1-OVA induced the highest percentage of CD4^+^DO11.10^+^ cells on day 3 after vaccination (**Figure 1D**). CD4^+^DO11.10^+^ cells were then analyzed for expression of T-bet, GATA-3, or RORγt, indicative of Th1, Th2, or Th17 cells, respectively. Xcl1-OVA induced a high percentage of T-bet^+^DO11.10 cells already at day 3 after immunization, which dropped off on days 5 and 7 after immunization, probably reflecting egress of T cells from the LN (**Figure 1E**). However, there were still ~10% T-bet^+^DO11.10 cells remaining at days 5 and 7 after immunization (**Figure 1E**). While Xcl1-OVA also induced a higher percentage of GATA3^+^ and RORγt^+^ cells 3 days after immunization, the numbers declined at 5 and 7 days after immunization (**Supplementary Figures 2C, D**). Consequently, DNA immunization with Xcl1-OVA induces expansion of T-bet^+^ cells with a clear Th1 phenotype within 1 week. In contrast, there was hardly any enhanced proliferation of DO11.10 cells after DNA immunization with fliC-OVA, although we did observe a slight increase on day 7 after immunization. To ensure that the OVA antigen did not adversely affect the antibody polarization, serum samples were analyzed for OVA-specific IgG1 and IgG2a antibody titers 2 weeks after i.d. DNA immunization with Xcl1- or fliC-OVA. As seen with fliC-HA, fliC-OVA induced an almost exclusive IgG1 response, while Xcl1 induced higher titers of IgG2a (**Supplementary Figure 2E**).

### Xcl1-OVA Fusion Proteins Enhance Th1 Polarization *In Vitro* and *In Vivo*

**Figure 2:**
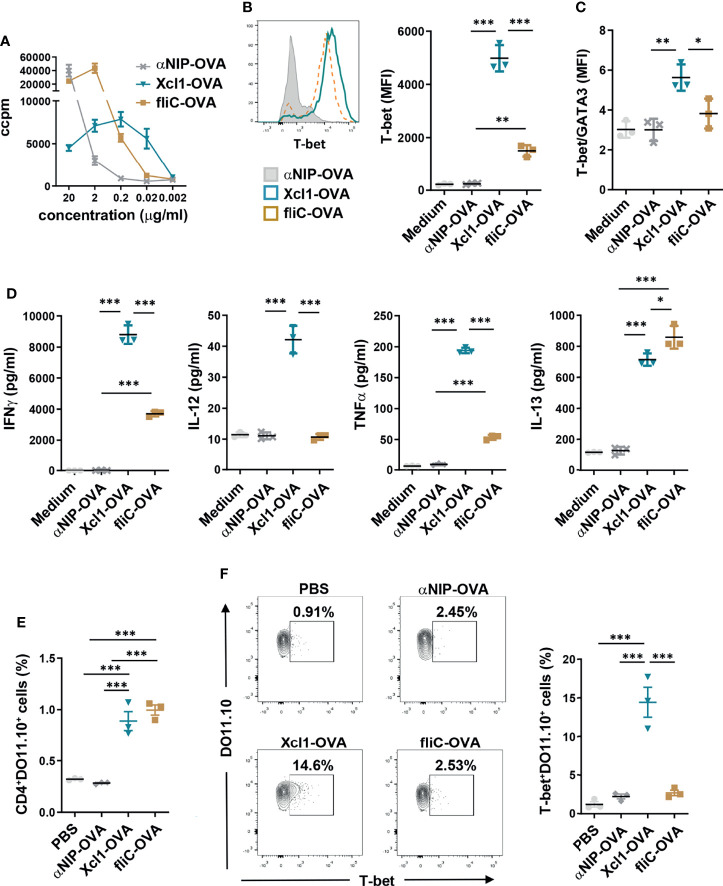
Xcl1-OVA induces Th1 polarization of DO11.10 cells *in vitro* and *in vivo*. **(A)** CD4^+^ cells were purified from spleen of DO11.10 TCR transgenic mice and incubated with BMDCs as APC in the presence of indicated amounts of Xcl1, fliC, or NIP-OVA protein for 72 h. Incorporation of radioactive thymidine was analyzed after 48 h. **(B–D)** CD4^+^ from DO11.10 transgenic mice were incubated with BMDC in the presence of Xcl1-, fliC-, or NIP-OVA proteins (0.5 μg/ml) for 72 h. **(B)** CD4^+^DO11.10^+^ cells were evaluated for expression of T-bet by flow cytometry. MFI for T-bet expression is summarized in the right graph. **(C)** Ratio of T-bet/GATA3 MFI for CD4^+^DO11.10^+^ cells in mice from **(B)** and **Supplementary Figure 3A**. **(D)** Concentrations of IFNγ, IL-12, TNFα, and IL-13 in supernatants. **(E, F)** 1 × 10^6^ naïve DO11.10 cells were transferred to BALB/c mice that were subsequently injected i.v. with purified Xcl1-OVA, fliC-OVA, or αNIP-OVA proteins (5 μg). Spleens were harvested 72 h later and the percentage of **(E)** CD4^+^DO11.10^+^ and **(F)** T-bet^+^DO11.10^+^ cells determined by flow cytometry. Data are representative of one **(A)** or three **(B–D)** independent experiments with n = 3 samples per group. **(E, F)** Data from one experiment with n = 3 mice per group. Statistical analysis was performed by one-way ANOVA with Tukey’s multiple-comparison corrections. *p < 0.05, **p < 0.01, ***p < 0.001.

The observation that i.d. DNA vaccination with fliC-OVA induced poor proliferation of CD4^+^ T cells *in vivo* was surprising. To test if this observation was related to the use of DNA, bone marrow-derived DCs (BMDCs) were incubated with DO11.10 cells and purified Xcl1- or fliC-OVA proteins in various concentrations for 72 h. In addition, we also included anti-NIP-OVA (referred to as αNIP) to serve as non-targeted control. Proliferation of the OVA-specific CD4^+^ T cells was determined by evaluating incorporation of radioactive thymidine (**Figure 2A**). Interestingly, purified fliC-OVA induced strong proliferation of DO11.10 cells, which was significantly higher than the non-targeted αNIP-OVA at 2 and 0.2 μg/ml. In contrast, Xcl1-OVA induced significantly higher proliferation than αNIP-OVA at concentrations <2 μg/ml and higher than fliC-OVA at 0.02 μg/ml (**Figure 2A**).

To study CD4^+^ T cell polarization mediated by Xcl1- or fliC-fusion vaccines, DO11.10 cells were incubated with BMDCs in the presence of 0.5 μg/ml Xcl1-OVA, fliC-OVA, or αNIP-OVA for 72 h. 0.5 μg/ml was chosen since this concentration induced similar proliferation with fliC- and Xcl1-OVA (**Figure 2A**). After 72 h, DO11.10 cells incubated with Xcl1-OVA displayed significantly higher expression levels of T-bet compared to αNIP-OVA- or fliC-OVA-incubated cells (**Figure 2B**). As seen after DNA vaccination *in vivo*, Xcl1-OVA also induced higher levels of GATA3 and RORγt compared to fliC-OVA, although the difference was lower than for T-bet (**Supplementary Figure 3A**). Indeed, when calculating the T-bet/GATA3 ratio, Xcl1-OVA clearly stood out as the strongest inducer of T-bet (**Figure 2C**). In accordance with the upregulation of T-bet, the supernatant from cells incubated with Xcl1-OVA contained significantly higher levels of the Th1-associated cytokines IFNγ, IL-12, and TNFα compared to supernatants from cells incubated with either fliC-OVA or αNIP-OVA (**Figure 2D**). DO11.10 cells incubated with fliC-OVA did not induce a clear polarization toward any Th subset based on the expression of T-bet, GATA-3, or RORγt (**Figures 2B, C** and **Supplementary Figure 3A**), although fliC-OVA induced slightly higher levels of IL-13 compared to Xcl1-OVA (**Figure 2D**). No difference was observed between Xcl1-OVA and fliC-OVA when determining secretion of the Th17-associated cytokine IL-17A, despite Xcl1-OVA inducing a higher expression of RORγt (**Supplementary Figures 3A, B**).

To obtain a better understanding on which DC subsets are presenting antigen to the DO11.10, sorted BM-derived cDC1s and cDC2s were incubated with CTV-labeled DO11.10 cells and 1 μg/ml Xcl1-OVA, fliC-OVA, or αNIP-OVA for 4 days. As expected, Xcl1-OVA predominantly induced proliferation of DO11.10 cells when incubated with cDC1s (**Supplementary Figures 3C, D**). While fliC-OVA induced significantly higher proliferation of DO11.10 cells on cDC2s compared to Xcl1-OVA, we were surprised to see that fliC-OVA also induced proliferation when incubated with cDC1s (**Supplementary Figures 3C, D**). Whether this is due to low-level TLR5 expression on BM cDC1s or activation of the NLRC4–NAIP5 inflammasome remains to be determined.

To test proliferation and polarization *in vivo*, 1 × 10^6^ CD4^+^DO11.10 cells were transferred to naïve BALB/c mice that were injected i.v. 1 day later with 5 μg purified Xcl1-, fliC-, or αNIP-OVA protein. After 3 days, spleens were harvested and single-cell suspensions analyzed by flow cytometry. Immunization with both Xcl1-OVA and fliC-OVA significantly enhanced the proliferation of DO11.10 cells, compared to αNIP-OVA- or PBS-immunized mice (**Figure 2E**). Correlating with our observations from DNA vaccination, immunization with Xc1-OVA protein induced a significantly higher percentage of T-bet^+^DO11.10 cells, compared to fliC-OVA and αNIP-OVA (**Figure 2F**). No increase in T-bet^+^DO11.10 cells was observed after immunization with fliC-OVA, although we did observe a slight but significant increase in the number of GATA3^+^DO11.10^+^ cells compared to αNIP-OVA (**Supplementary Figure 4A**). There was no difference in the percentage of RORγt^+^ DO11.10 cells in mice immunized with Xcl1-OVA or fliC-OVA, although there was a slight increase for both compared to αNIP-OVA (**Supplementary Figure 4B**).

### Xcl1-OVA-Induced Th1 Polarization Is Dependent on IL-12, but Independent of BATF3

**Figure 3:**
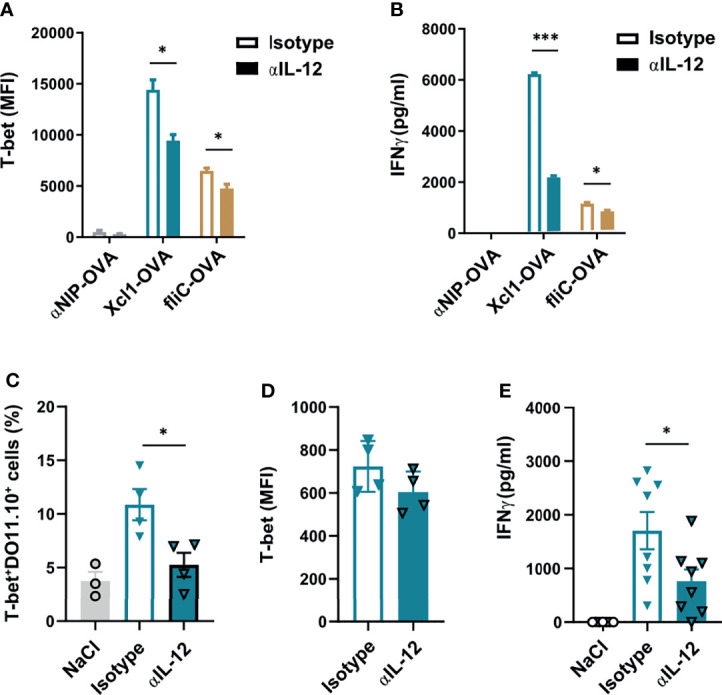
Xcl1-OVA induced Th1 polarization is IL12 dependent. **(A, B)** CD4^+^ cells from DO11.10 mice were incubated with BMDCs and Xcl1-OVA, fliC-OVA, or αNIP-OVA proteins (0.5 μg/ml), and either anti-IL-12 or isotype-matched mAbs (10 μg/ml) for 72 h. **(A)** DO11.10 cells were evaluated for expression of T-bet by flow cytometry, and **(B)** supernatants tested for secretion of IFNγ by ELISA. **(C–E)** 1 × 10^6^ naïve DO11.10 cells were transferred i.v. to BALB/c mice that were subsequently immunized i.d. with 25 μg DNA encoding Xcl1-OVA. On days 1 and 2 after immunization, mice were injected i.p. with anti-IL12 or isotype-matched mAb (0.5 mg). Skin draining LNs and spleens were harvested after 1 week, and LN analyzed for **(C)** percentage of T-bet^+^DO11.10^+^ cells and **(D)** MFI of T-bet expression in T-bet^+^DO11.10^+^ cells. **(E)** Secretion of IFNγ from splenocytes stimulated for 24 h with the DO11.110 peptide. Data representative of two **(A–D)** or pooled from two **(E)** independent experiments with n = 3 samples per group **(A, B)**, n = 3–4 mice per group **(C, D)** or n = 7–8 mice per group **(E)**. Statistical analysis performed using the parametric t-test. *p < 0.05, ***p < 0.001.

Targeting antigens to the lectin receptor DEC-205 expressed on cDC1s has been reported to induce IL-12-independent Th1 responses through upregulation of CD70 (34). To evaluate the role of IL-12 in induction of Th1 responses when targeting cDC1s using Xcl1, DO11.10 cells were incubated with BMDCs and 0.5 μg/ml Xcl1-OVA, fliC-OVA, or αNIP-OVA protein in addition to the anti-IL-12 antibody. Blocking of IL-12 resulted in a significant reduction in T-bet expression in DO11.10 cells incubated with both Xcl1-OVA and fliC-OVA (**Figure 3A**). Correlating with reduced expression of T-bet, there was a significant reduction in the secretion of IFNγ (**Figure 3B**). Incubation with Xcl1-OVA in the presence of anti-IL-12 did not significantly influence the expression of GATA3 or the secretion of IL-4 (**Supplementary Figures 5A, B**).

To explore the IL-12 dependency of Xcr1-targeted Th1 responses *in vivo*, we transferred 1 × 10^6^ DO11.10 cells to naïve BALB/c mice which were then immunized with 25 μg DNA encoding for Xcl1-OVA i.d 24 h later. The mice were subsequently injected i.p. with either 0.5 mg anti-IL-12 or isotype-matched control 24 and 48 h after vaccination. Skin-draining LNs and spleens were then harvested on day 7 after vaccination. Single-cell suspensions from the LNs were analyzed by flow cytometry, while splenocytes were restimulated with 2 μg/ml of the OVA_323-339_ peptide for 48 h and secretion of IFNγ was analyzed by ELISA. Interestingly, there was a significant decline in the expansion of T-bet^+^ DO11.10 cells in mice treated with anti-IL-12 (**Figure 3C**). The expression level of T-bet was however comparable between anti-IL-12-treated and control mice (**Figure 3D**). In accordance with fewer Th1 cells, there was a significant drop in the secretion of IFNγ from mice that were injected with anti-IL-12 antibodies compared with isotype control mice (**Figure 3E**). Taken together, our results suggest that the expansion of Th1 cells after i.d. DNA vaccination with Xcl1-OVA is IL-12 dependent.

**Figure 4:**
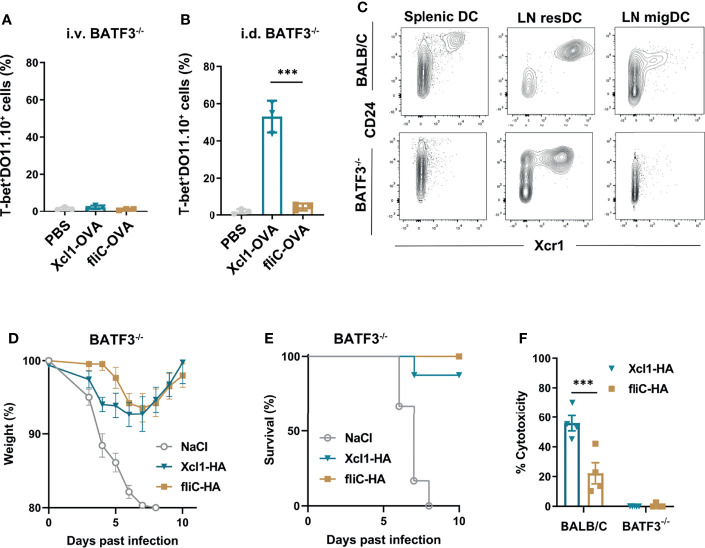
Xcl1-OVA induced Th1 polarization is independent of BATF3. **(A, B)** BATF3^-/-^ mice were injected i.v. with 1 × 10^6^ naïve DO11.10 cells, and 24 h later **(A)** given an i.v. injection of either purified Xcl1- or fliC-OVA proteins (5 μg) or **(B)** DNA immunized i.d. with 25 μg plasmid encoding either Xcl1-OVA or fliC-OVA. The percentages of T-bet^+^ DO11.10 cells evaluated in spleens **(A)** or in LNs **(B)** 3 days after immunization. **(C)** Spleens and inguinal LNs were harvested from BALB/c or BATF3^-/-^ mice and analyzed for CD24^+^Xcr1^+^ cDC1s by flow cytometry after first gating on Lin^-^MHC-II^+^CD11c^+^ cells. Migratory DCs were defined as CD11c^int^MHC-II^high^ and resident DCs as CD11c^high^MHC-II^int^ (**Supplementary Figure 5F**). **(D, E)** BATF3^-/-^ mice were DNA immunized with 25 μg plasmid encoding either Xcl1-OVA or fliC-OVA and challenged 14 days later with 5xLD50 PR8 virus. **(D)** Weight and **(E)** survival was monitored for 10 days. **(F)** *In vivo* cytotoxicity after DNA immunization with 25 μg plasmid encoding either Xcl1-OVA or fliC-OVA in BALB/c or BATF3^-/-^ mice. Data representative of two **(A–C)**, pooled from two **(D, E)** or from one **(F)** independent experiments with n = 3 samples per group **(A, B)**, n = 3–4 mice per group **(C, F)** or n = 6–8 mice per group **(D, E)**. Statistical analysis performed using parametric t-test. ***p < 0.001.

BATF3 is a transcription factor that is essential for the development of Xcr1^+^ cDC1s in spleen and CD103^+^Xcr1^+^ migratory cDC1s in skin-draining LN (35). Previous studies using different infectious models have suggested that the BATF3-dependent migratory CD103^+^ cDC1s are the main producers of IL-12 that drive Th1 polarization (30). To test how the absence of BATF3 impacted the observed Th1 polarization seen with Xcl1-OVA, BATF3^-/-^ were immunized with Xcl1-OVA, fliC-OVA, or αNIP-OVA by i.v. injection of purified protein and by i.d. DNA immunization. After i.v. injection of protein, fliC-OVA induced the proliferation of DO11.10 cells in spleen, potentially by targeting the BATF3-independent cDC2 population (**Supplementary Figure 5C**). In contrast, we observed no proliferation or induction of T-bet^+^ DO11.10 cells with Xcl1-OVA after i.v. injection (**Figure 4A** and **Supplementary Figure 5C**). Surprisingly, DNA immunization with Xcl1-OVA induced a strong increase in the number of CD4^+^DO11.10^+^ and T-bet^+^ DO11.10 cells in the BATF3^-/-^ mice (**Figure 4B** and **Supplementary Figure 5D**). Indeed, the frequency of T-bet^+^ DO11.10 cells was similar to those seen in BALB/c mice (**Figure 2F**), indicating that Th1 polarization after i.d. DNA vaccination with Xcl1-OVA is BATF3-independent. In support of this observation, i.d. DNA immunization with fliC-HA or Xcl1-HA in BATF3^-/-^ mice induced a similar IgG1 to IgG2a-polarized antibody response as in BALB/c mice (**Supplementary Figure 5E**).

To investigate the observed difference between i.v. protein and i.d. DNA vaccination with Xcl1-OVA, we analyzed Xcr1 expression on MHC-II^+^CD11c^+^ DCs from spleen and skin-draining LN after i.d. DNA immunization. In skin-draining LN, migratory DCs (migDCs) were defined as CD11c^int^MHC-II^high^, while resident DCs (resDCs) were defined as CD11c^high^MHC-II^int^. While CD24^+^Xcr1^+^ DCs were absent in the spleen of BATF3^-/-^ mice, we observed a clear population of CD24^+^Xcr1^+^ resDCs in inguinal LNs from the same mice (**Figure 4C** and **Supplementary Figure 5F**). However, we did not observe any CD24^+^ Xcr1^+^ migDC in the LN from BATF3 KO mice, which is in accordance with previous observations (35). The presence of Xcr1^+^ resident DCs in LN provides a possible explanation of why i.d. DNA immunization with Xcl1-OVA still induces CD4^+^ T cell proliferation and Th1 polarization in the BATF3^-/-^ mice.

To evaluate if the immune responses seen in BATF3^-/-^ mice was sufficient to mediate protection against influenza infection, BATF3^-/-^ mice were DNA immunized with Xcl1-HA or fliC-HA and challenged with 5xLD50 2 weeks later. Both Xcl1-HA- and fliC-HA-immunized mice were protected from challenge and only displayed moderate weight loss during the infection (**Figures 4D, E**). As BATF3^-/-^ mice do not induce cytotox T cell responses after DNA vaccination (**Figure 4F**), this observation suggests that protection seen with Xcl1-HA is predominantly mediated by the antibody response.

### Xcl1-Fusion Vaccines Maintain Th1/IgG2a Polarization When Combined With fliC-Fusion Vaccines *In Vitro* and *In Vivo*

Our results suggest that while Xcl1-fusion vaccines enhance Th1 polarization *in vitro* and *in vivo*, the fliC-fusion vaccines had a more modest Th2 polarization *in vitro.* The lack of a clear Th2 polarization with fliC-fusion vaccines is surprising given the highly IgG1-polarized antibody response seen after DNA vaccination with fliC-HA. fliC has previously been described to actively inhibit Th1 polarization (36), which could explain why fliC-OVA did not induce Th1 responses despite inducing proliferation of DO11.10 cells when incubated with cDC1s. To test if this was the case, DO11.10 cells were stimulated with BMDCs and a mixture of Xcl1-OVA and fliC-OVA. The Xcl1-OVA/fliC-OVA mix induced lower levels of T-bet compared to Xcl1-OVA alone, although the difference was not significant, and probably reflects the fact that half the concentration of Xcl1-OVA was present in the mix (**Supplementary Figure 6A**). There was also a slight reduction in GATA3 expression with the mix compared to Xcl1-OVA alone (**Supplementary Figure 6A**). When evaluating cytokines expressed in supernatants, the Xcl1-OVA/fliC-OVA mix induced equal levels of IFNγ, and lower levels of IL-12 compared to Xcl1-OVA (**Supplementary Figure 6B**). Together, these results indicate that the Th1 polarization is largely maintained in the mix and not actively inhibited by the fliC-fusion protein.

**Figure 5:**
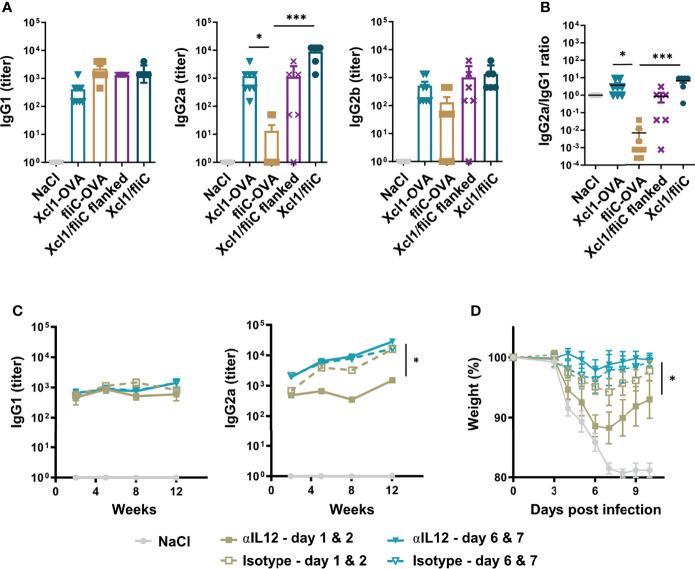
Rapid Th1 induction is essential for induction of an IgG2a dominated response and improves protection induced by a mixed fliC-/Xcl1-HA vaccine. **(A, B)** DNA immunization of BALB/c mice with 25 μg DNA encoding Xcl1-HA, fliC-HA, a mixture of Xcl1-HA and fliC-HA, or Xcl1-HA and fliC-HA delivered on opposite flanks. **(A)** Serum titers of IgG1, IgG2a, and IgG2b were determined 2 weeks after vaccination. **(B)** IgG2a/IgG1 ratio of the serum samples presented in **(A)**. **(C, D)** Injection of either anti-IL-12 or isotype-matched mAbs on days 1 and 2 or 6 and 7 after DNA immunization with a mix of Xcl1-HA and fliC-HA plasmids. **(C)** Serum samples were harvested at the indicated time points and evaluated for the presence of HA-specific IgG1 or IgG2a. **(D)** Mice in **(C)** were challenged with 5xLD50 PR8 virus 12 weeks after immunization and weight loss monitored. Data representative of one experiment with n = 6–8 mice per group **(A, B)**, representative of two independent experiments with n = 4 mice per group **(C)** or pooled from two independent experiments with n = 8 mice per group **(D)**. Statistical analysis performed by non-parametric one-way ANOVA with Dunn’s multiple-comparison corrections **(A, B)**, or two-way ANOVA with Tukey’s multiple-comparison test **(C, D)**. *p < 0.05, ***p < 0.001.

Next, we tested if Xcl1-HA was able to skew the antibody response in the direction of IgG2a *in vivo* when combined with fliC-HA and delivered by i.d. DNA vaccination. As controls, BALB/c mice were immunized with Xcl1-HA and fliC-HA separately, or Xcl1-HA and fliC-HA delivered on opposite flanks of the mouse. Immunization on opposite flanks should predominantly result in draining and T cell priming in separate inguinal LNs. Serum samples were harvested after 2 weeks and evaluated for the presence of HA-specific antibodies of the IgG1, IgG2a, and IgG2b subclasses. As observed in **Figure 1**, immunization with Xcl1-HA or fliC-HA induced antibody responses dominated by IgG2a/IgG2b and IgG1, respectively (**Figure 5A**). Immunization with a mix of Xcl1-HA and fliC-HA induced similar levels of IgG1 as fliC-HA alone but significantly higher titers of IgG2a. Consequently, the IgG2a/IgG1 ratio seen with the mix was similar to that of Xcl1-HA alone (**Figure 5B**). In contrast, immunization with Xcl1-HA and fliC-HA on separate flanks induced similar titers of IgG1 and lower titers of IgG2a compared to the mix, resulting in a lower IgG2a/IgG1 ratio (**Figures 5A, B**). Together, these results suggest that the Xcl1-fusion vaccine exerts dominance in determining the polarization of the antibody response.

### A Rapid Th1 Response Is Required for an IgG2a-Dominated Response and Contributes to Protective Responses

To further test how the kinetics of the Th1 response influence antibody polarization, we immunized mice with a mix of Xcl1-HA and fliC-HA and subsequently injected the anti-IL-12 or isotype antibody i.p. on days 1 and 2 or days 6 and 7 after immunization. The mix vaccine was chosen as it induced the strongest antibody responses with similar polarization to Xcl1-HA. Serum samples were harvested after 2, 5, 8, and 12 weeks after immunization and evaluated for HA-specific antibodies of the IgG1, IgG2a, and IgG2b subclasses. Early injection of anti-IL-12 resulted in a significant reduction in IgG2a and IgG2b titers at 5, 8, and 12 weeks after immunization (**Figure 5C**, **Supplementary Figure 6C**). In contrast, there was no difference in IgG2a when anti-IL-12 was injected on days 6 and 7 after immunization (**Figure 5C**). There was also no difference in the HA-specific IgG1 titer after 5, 8, or 12 weeks, suggesting that injection of anti-IL-12 only affected Th1-associated IgG subclasses. Interestingly, early injection of anti-IL-12 resulted in more dramatic weight loss after influenza infection after 12 weeks, compared to injection of anti-IL-12 on days 6 and 7 after immunization (**Figure 5D**). There was also a reduction in the overall survival, although the difference was not significant (**Supplementary Figure 6D**). These results suggest that the early Th1 response is important for obtaining a strong IgG2a response and that Th1 cells contribute to the protection seen with the Xcl1/fliC vaccine.

In summary, our observations indicate that antibody polarization after DNA vaccination is determined very early after immunization and that Xcl1 fusion vaccines preferentially induce IgG2a and IgG2b due to a rapid induction of Th1 cells.

## Discussion

Here we compare Xcl1- and fliC-fusion vaccines in terms of the ability to differently influence the polarization of the resulting immune response. While Xcl1-fusion vaccines rapidly polarize CD4^+^ T cells toward Th1 after immunization, fliC-fusion vaccines induced a more mixed Th1/Th2 polarization despite inducing almost exclusively antibodies of the IgG1 subclass. We further demonstrate that inhibiting Th1 polarization early after DNA immunization significantly reduced IgG2a and IgG2b responses, resulting in poorer protection against influenza infection. The results suggest that early induction of Th1 responses is a key determining factor in the polarization of the antibody response.

Previous studies have suggested that cDC1s and cDC2s preferentially polarize CD4^+^ T cells toward Th1 and Th2, respectively (9, 10). Indeed, our observations that Xcl1-fusion proteins induce IgG2a-dominated antibody responses and Th1 polarization support these findings (13, 19, 29, 31). However, we have recently observed that the choice of target-receptor on cDC1s can influence the resulting immune response (19). It is therefore possible that the Xcl1–Xcr1 ligand interaction could lead to downstream signaling events in the cDC1s that enhance Th1 responses. Indeed, early studies have suggested that Xcl1 functions in concert with IFNγ, MIP1α, MIP1β, and RANTES in promoting Th1 responses after infection (20). Although fliC-OVA also induced the proliferation of DO11.10 cells when incubated with cDC1s *in vitro*, it did not induce any significant Th1 polarization in the *in vitro* or the *in vivo* experiments. This indicate that additional stimulation of the cDC1s is needed to induce Th1 polarization, although it is currently not clear if fliC-fusion proteins target cDC1s *in vivo* to any significant degree.

Our experiments suggest that the early kinetics of the Th1 responses is crucial in obtaining an IgG2a-polarized antibody response. Class switch recombination (CSR) has been considered to occur within the germinal center (GC) reaction in combination with affinity maturation through somatic hyper mutation (SHM) (37). However, a recent study by Roco et al. observed that CSR predominantly takes place prior to GC formation and largely within 3–4 days of antigen challenge (38). Here we observe that injecting anti-IL-12 on days 1 and 2 after i.d. DNA vaccination with a Xcl1/fliC-HA mix significantly reduced the induction of IgG2a. In contrast, delaying anti-IL-12 injection until days 6 and 7 did not influence the IgG2a titers. Our experiments therefore correlate with a rapid CSR through induction of Th1 cells, which should be taken into consideration when developing vaccines aimed at inducing Th1-polarized immune responses.

These experiments also indicate that IL-12 played an important role in inducing efficient Th1 polarization and IgG2a responses after intradermal DNA immunization with Xcl1 fusion vaccines. These observations are contrary to previous studies by Soares and colleagues where targeting the LACK antigen from *Leishmania major* to DEC-205 (CD205) expressed on cDC1s resulted in an IL-12-independent induction of Th1 responses (34). However, it should be noted that these experiments were performed with addition of poly(I:C) as an adjuvant, which activates TLR3 expressed on cDC1s (25). Indeed, older studies have observed that viral infections with RNA viruses, which can trigger TLR3 activation, also induce an IL-12-independent induction of Th1 CD4^+^ T cell and IgG2a responses (39).

Interestingly, i.d. DNA vaccination with Xcl1-OVA induced equal Th1 polarization and IgG2a induction in BATF3^-/-^ mice. These mice have been reported to lack IL-12-producing CD103^+^ cDC1s, disrupting their ability to induce Th1 responses in response to *Leishmania major* infection (30). In our study, flow cytometry analysis of skin-draining LN demonstrated the presence of Xcr1^+^ resDCs in the BATF3^-/-^ mice, while the Xcr1^+^ migDC population was absent. These results are in accordance with observations by Bachem and colleagues (35) and may suggest that Xcr1^+^ resDCs are responsible for inducing the rapid Th1 responses after intradermal DNA immunization with Xcl1-fusion vaccines. However, specific depletion of these cells would be required to confirm this hypothesis.

DNA immunization of BATF3^-/-^ did not give any cytotoxic T cell responses, although the mice were still protected from a lethal challenge with influenza virus. Consequently, the antibodies induced after one immunization with Xcl1-HA or fliC-HA were sufficient to protect against infection. In addition, our results may indicate that the Xcr1^+^ migDC population is needed for the induction of cytotoxic CD8^+^ T cell responses after DNA vaccination (31). However, it is also possible that lack of BATF3 directly influences the cytotoxic function of the CD8^+^ T cells, as recent studies have shown that BATF3 regulates the formation of CD8^+^ memory T cells (40, 41).

We have previously observed that XCL1-fusion proteins can bind cDC1s from humans (42), macaques (42), and pigs (16, 43), as expression of the XCR1 receptor appear to be largely conserved on cDC1s in mammals (44). Consequently, Xcl1-fusion may be utilized in both clinical and veterinary medicine. However, it is currently unclear if our observations that Xcl1-fusion vaccines enhance Th1 polarization can be translated to other species. For instance, both human cDC1s and cDC2s can secrete IL12 and induce Th1 polarization (45, 46), raising the question whether there is any added effect of human XCR1–XCL1 ligation.

FliC-fusion vaccines only induced antibodies of the IgG1 subclass after i.d. DNA vaccination, despite limited proliferation and polarization of DO11.10 cells in iLNs. The lack of T cell proliferation after DNA vaccination *in vivo* was surprising given the observation that fliC-OVA could induce T cell proliferation when incubated with purified cDC1s and cDC2s. We did however observe increased the numbers of IFNγ-secreting CD4^+^ T cells 2 weeks after immunization by ELISPOT analysis. *In vitro*, the purified fliC-OVA protein induced a more mixed Th1/Th2 polarization, although we did observe a modest upregulation of GATA3 *in vivo*. Consequently, our observations are in line with previous studies suggesting that fliC can induce a mixed Th1/Th2 responses (47, 48), instead of a pronounced Th2 polarization (21). It is possible that the responses obtained with fliC-fusion vaccines are dependent on other cell types than cDC2s, as TLR5 has also been reported to be expressed on pDCs and CD8^+^Xcr1^-^ DCs in skin-draining LN (24, 25). Indeed, when evaluating TLR5 expression on DCs in spleen, we observed a low-level expression on both cDC1s and cDC2s. It is however unlikely that fliC directly induces class switch recombination to IgG1, considering that TLR5 has been reported to be absent from murine B cells (49).

In conclusion, our results demonstrate that IgG2a/IgG2b polarization of the antibody responses is determined early after immunization, which should be taken into account when designing or evaluating immunization strategies aimed at inducing specific subclasses of IgG. For instance, non-neutralizing mAbs against influenza HA have been reported to provide protection when injected as IgG2a through Fc-mediated effector function, but not when injected as IgG1 (50).

## Data Availability Statement

The original contributions presented in the study are included in the article/**Supplementary Material**. Further inquiries can be directed to the corresponding author.

## Ethics Statement

All animal studies were reviewed and approved by Norwegian Food Safety Authority.

## Author Contributions

DT, SB, AL, EF, and BB designed the experiments and conceptualized the study. DT, SB, AL, AG, PCH, and RB performed and analyzed the experiments. EF, DT, and BB wrote the first draft of the manuscript. All authors contributed to the article and approved the submitted version.

## Funding

This study was funded by KG Jebsen Foundation (KG Jebsen Foundation, BB), EuroNanoMed (EU Grant 2012023, BB), Norway, Regional Health Authority (HSØ grant 2019118, EF) and The Research Council of Norway (NFR, Grant 250884, EF).

## Conflict of Interest

BB is the inventor on patents on the vaccine molecules described herein (Vaccibodies). He is the leader of the scientific panel of Vaccibody AS and has shares in the company.

The remaining authors declare that the research was conducted in the absence of any commercial or financial relationships that could be construed as a potential conflict of interest.

## Publisher’s Note

All claims expressed in this article are solely those of the authors and do not necessarily represent those of their affiliated organizations, or those of the publisher, the editors and the reviewers. Any product that may be evaluated in this article, or claim that may be made by its manufacturer, is not guaranteed or endorsed by the publisher.
