# Supplementary material for: Targeting Xcr1 on Dendritic Cells Rapidly Induce Th1-Associated Immune Responses That Contribute to Protection Against Influenza Infection
Source: Front Immunol. 2022 Feb 28;13:752714. doi: 10.3389/fimmu.2022.752714 (PMC8918470; doi:10.3389/fimmu.2022.752714)

Supplementary Figure Legends

Supplementary Figure 1: Characterization of Xcl1- and fliC-fusion vaccines. (a) Illustration of the dimeric fusion vaccine structure consisting of a targeting unit containing Xcl1 or fliC, a dimerization domain consisting of hinge and CH_3 from human IgG3 and an antigenic domain consisting of HA from influenza A/Puerto Rico/8/34 (PR8) or ovalbumin (OVA). (b) BM DCs were defined as $CD45R^-CD11c^+$ cells and further divided into $CD24^+$cDC1s and $CD11b^+$cDC2s. Expression of Xcr1 and TLR5 were analyzed on the cDC1 and cDC2 populations. (c) Splenic DCs were defined as $Lin^-MHC\text{-}II^+CD11c^+$ cells and divided into $CD24^+$ cDC1s and $CD11b^+$ cDC2s. TLR5 expression was evaluated on both DC subsets in comparison with an isotype control. (d) HA-specific IgG2b serum responses after i.d. DNA vaccination with 25μg plasmid encoding Xcl1-OVA or fliC-OVA followed by electroporation. (e-f) BALB/C mice were DNA immunized once as in (d) and subsequently challenged with 50xLD50 influenza A virus (PR8) after 2 weeks. (e) Weight loss was monitored over time as a sign of disease progression. (f) Survival plot of mice presented in (e). Data are representative of one (c) or two independent experiments (b) or pooled from 2 independent experiments (d-f), with n = 20 (d) or n = 10 (e-f) mice pr group. Statistical analysis performed using non-parametric t-test (c). *** = $p < 0.001$.

Supplementary Figure 2: Xcl1-fusion vaccines induces rapid Th1 responses after DNA vaccination. (a-b) IFNγ and IL4 ELISPOT on splenocytes harvested from BALB/C mice (a) 1 or (b) 2 weeks after i.d. DNA vaccination with 25μg plasmid encoding Xcl1-OVA or fliC-OVA followed by electroporation. Splenocytes were stimulated with the HA derived MHC-II restricted peptide HNTNGVTAACSHEG. (c-d) $1x10^6$ naïve DO11.10 cells were transferred to BALB/C mice that were subsequently immunized as in (a). Inguinal LNs were harvested 3, 5 or 7 days after vaccination and evaluated for (c) percentage of $GATA3^+$ and (d) percentage of $ROR\gamma t^+$ $CD4^+DO11.10^+$ cells. (e) Serum titers of OVA specific IgG1 and IgG2a from BALB/C mice 2 weeks after i.d. DNA immunization as in (a). Data shown are from one single experiment (a and e), pooled from two independent experiments (b) or representative of 2 independent experiments (c-d), with n = 6 mice pr group (a), n = 8 mice pr group (b), n = 3 mice pr group (c-d) or 2-4 mice pr group (e). Statistical analysis performed using parametric t-

test (a-b), one-way-anova with Tukey`s multiple comparison test comparing Xcl1-OVA and fliC-OVA for the different timepoints (c-d) or non-parametric t-test (e). * = $p < 0.05$, ** = $p < 0.01$, *** = $p < 0.001$.

Supplementary Figure 3: Polarization and proliferation of DO11.10 cells *in vitro*. a) $CD4^+$ cells were purified from spleens of DO11.10 TCR transgenic mice and incubated with BMDCs and either Xcl1-, fliC- or αNIP-OVA proteins (0.5 μg/ml) for 72h. DO11.10 cells were analyzed for expression of (a) GATA3 or RORγt by MFI. (b) Supernatants from cell cultures in (a) were evaluated for secretion of IL17A by ELISA. (c-d) Proliferation of CTV labelled $CD4^+$DO11.10 cells incubated with sorted BM derived cDC1s or cDC2 and 1 μg/ml Xcl1-OVA or fliC-OVA for 4 days. As a positive control, cells were incubated with 0.5 μg/ml of the $OVA_{323\text{-}339}$ peptide. (d) Summary of the proliferation data presented in (c). (a-b) Data representative of three independents experiments or (c-d) of one experiment with n = 3 samples pr group. Statistical analysis performed by one-way-anova with Tukey`s multiple comparison corrections. * = $p < 0.05$, ** = $p < 0.01$, *** = $p < 0.001$.

Supplementary Figure 4: Polarization of DO11.10 cells *in vivo.* (a-b) $1x10^6$ naïve DO11.10 cells were transferred to BALB/c mice that were subsequently immunized i.v. with 5μg purified Xcl1-OVA, fliC-OVA or αNIP-OVA protein. Spleens were harvested 72h later and the percentage $GATA3^+$ (a) and $ROR\gamma t^+$ (b) cells determined by flow cytometry after gating on $CD19^-CD3^+CD4^+DO11.10^+$ cells. Graphs on the right summarizes the data from 3 individual mice. Data representative of two independent experiments with n = 3 mice pr group. Statistical analysis performed by one-way-anova with Tukey`s multiple comparison corrections. * = $p < 0.05$, ** = $p < 0.01$, *** = $p < 0.001$.

Supplementary Figure 5: Evaluation of polarization after incubation with anti-IL-12, and BATF3 dependency after i.v. protein injection and i.d. DNA vaccination. (a-b) $CD4^+$ cells from DO11.10 mice were incubated with BMDCs and Xcl1-OVA, fliC-

OVA or αNIP-OVA proteins (0.5 μg/ml) and with either anti-IL-12 or isotype-matched mAbs (10 μg/ml) for 72h. (a) DO11.10 cells were evaluated for expression of GATA3 by flow cytometry, and (b) supernatants tested for secretion of IL-4 by ELISA. (c-d) $1x10^6$ naïve DO11.10 cells were transferred to BATF3$^{-/-}$ mice that were subsequently injected i.v. with 5μg purified Xcl1-OVA or fliC-OVA (c) or DNA vaccinated with 25μg plasmid encoding Xcl1-OVA or fliC-OVA (d). Spleens (c) or inguinal LNs (d) were harvested 72h later and the percentage of CD4$^+$DO11.10$^+$ cells determined by flow cytometry. (e) HA-specific IgG1 (left graph) and IgG2a (right graph) serum responses 2 weeks after i.d. DNA vaccination with 25μg plasmid encoding Xcl1-OVA or fliC-OVA followed by electroporation. (f) Gating strategy for separating migratory DCs (MigDC) and resident DCs (ResDC) in skin-draining LN. Data are representative of two independent experiments (a-b, d) or one experiment (c, e-f) with n = 3 samples pr group (a-b), n = 3 mice pr group (c-d,f) or n = 2-4 mice per group (e). Statistical analysis performed using parametric t-test (a-d) or Mann-Whitney test (e). * = $p < 0.05$, *** = $p < 0.001$.

Supplementary Figure 6: Xcl1-OVA maintains Th1 polarization when combined with fliC-OVA. (a-b) CD4$^+$DO11.10 cells were isolated and incubated with BMDCs in the presence of either Xcl1-OVA, fliC-OVA, αNIP-OVA protein (0.5 μg/ml) or a mix of Xcl1-OVA and fliC-OVA (0.25 μg/ml of each). (a) Cells were harvested after 72h and expression of T-bet and GATA3 evaluated by intracellular flow cytometry. (b) Supernatants from the stimulated DO11.10 cells in (a) were evaluated for IFNγ and IL-12 by ELISA. (c) Following DNA immunization with a mix of Xcl1-HA and fliC-HA, mice were injected with either anti-IL-12 or isotype-matched mAbs on day 1 and 2 or 6 and 7. Serum samples were harvested at the indicated time points and evaluated for the presence of HA-specific IgG2b. (d) Survival plot of mice in (c) after challenge with 5xLD50 PR8 virus 12 weeks after immunization. (a-b) Data are representative of two independent experiments with n = 3 samples per group, or (c-d) pooled from two independent experiments with n = 7-8 mice per group. Statistical analysis performed using one-way ANOVA with Tukey`s multiple comparison corrections (a-b), two-way-anova with Tukey`s multiple comparison test (c). * = $p < 0.05$, ** = $p < 0.01$, *** = $p < 0.001$.

Supplementary Figure 1

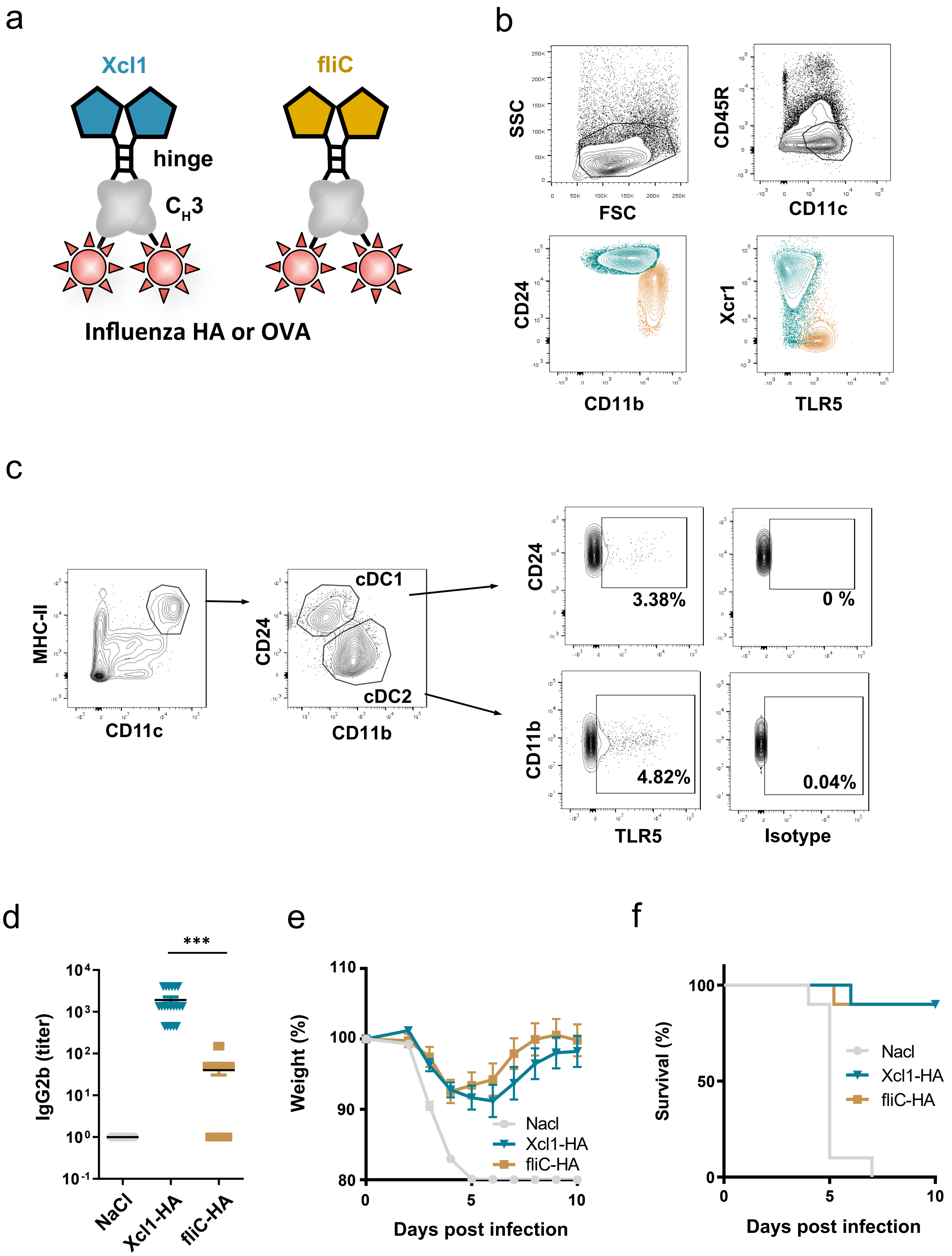

Supplemental Figure 2

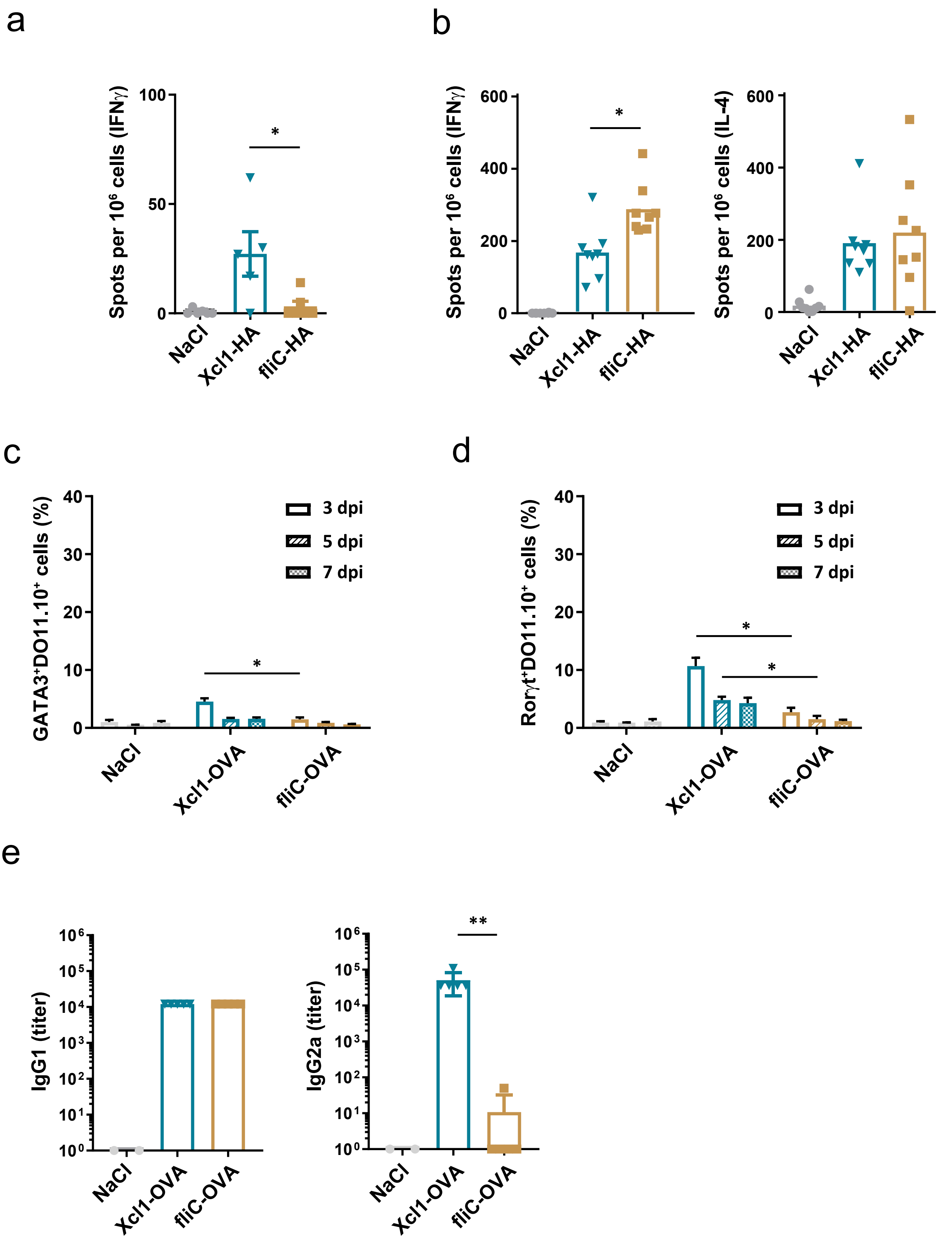

Supplementary Figure 3

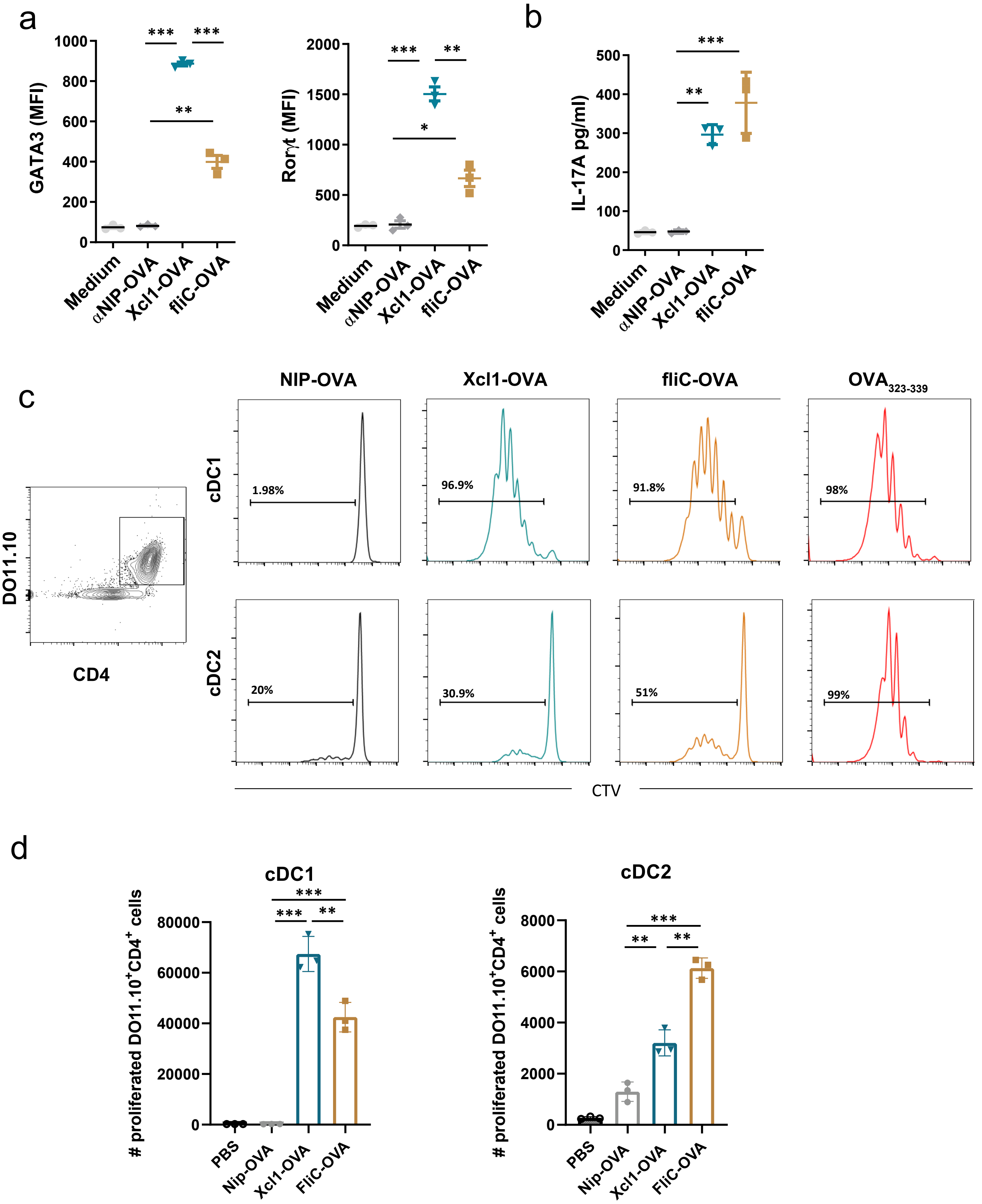

Supplementary Figure 4

a

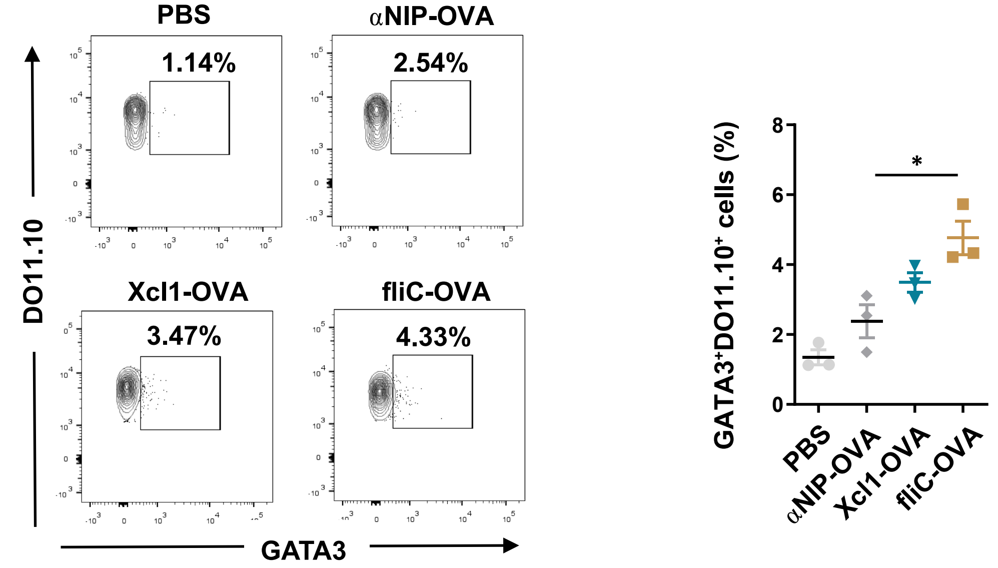

b

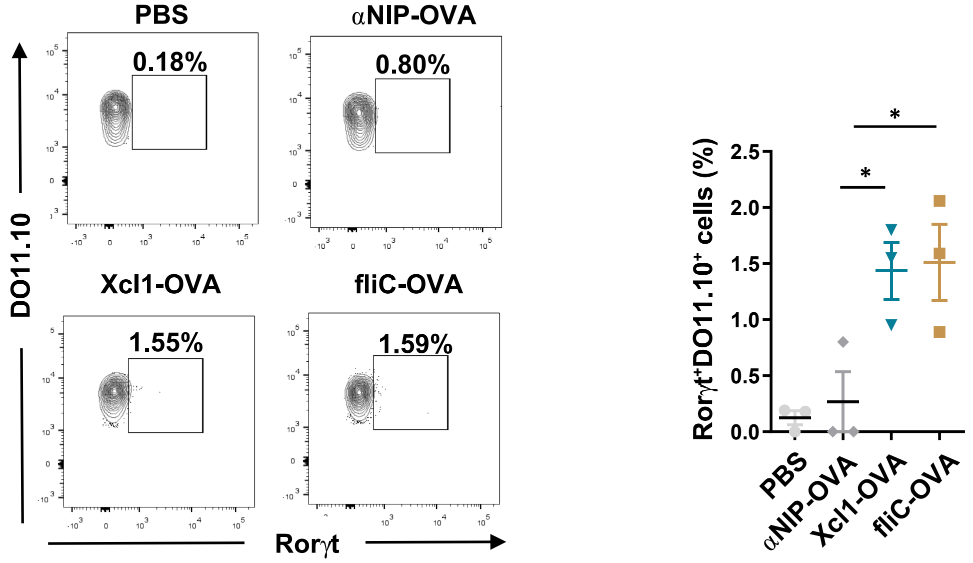

Supplementary Figure 5

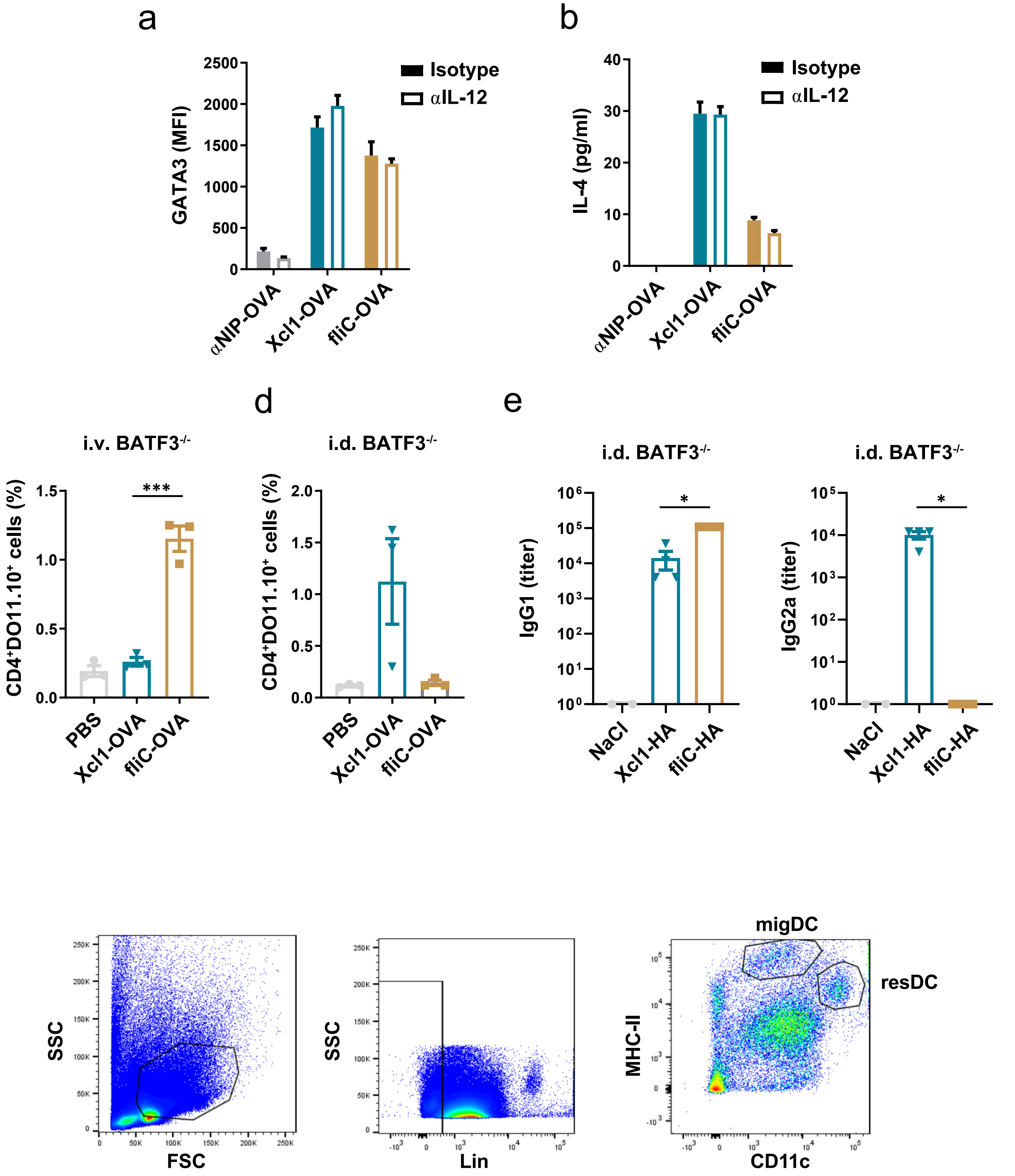

Supplementary Figure 6

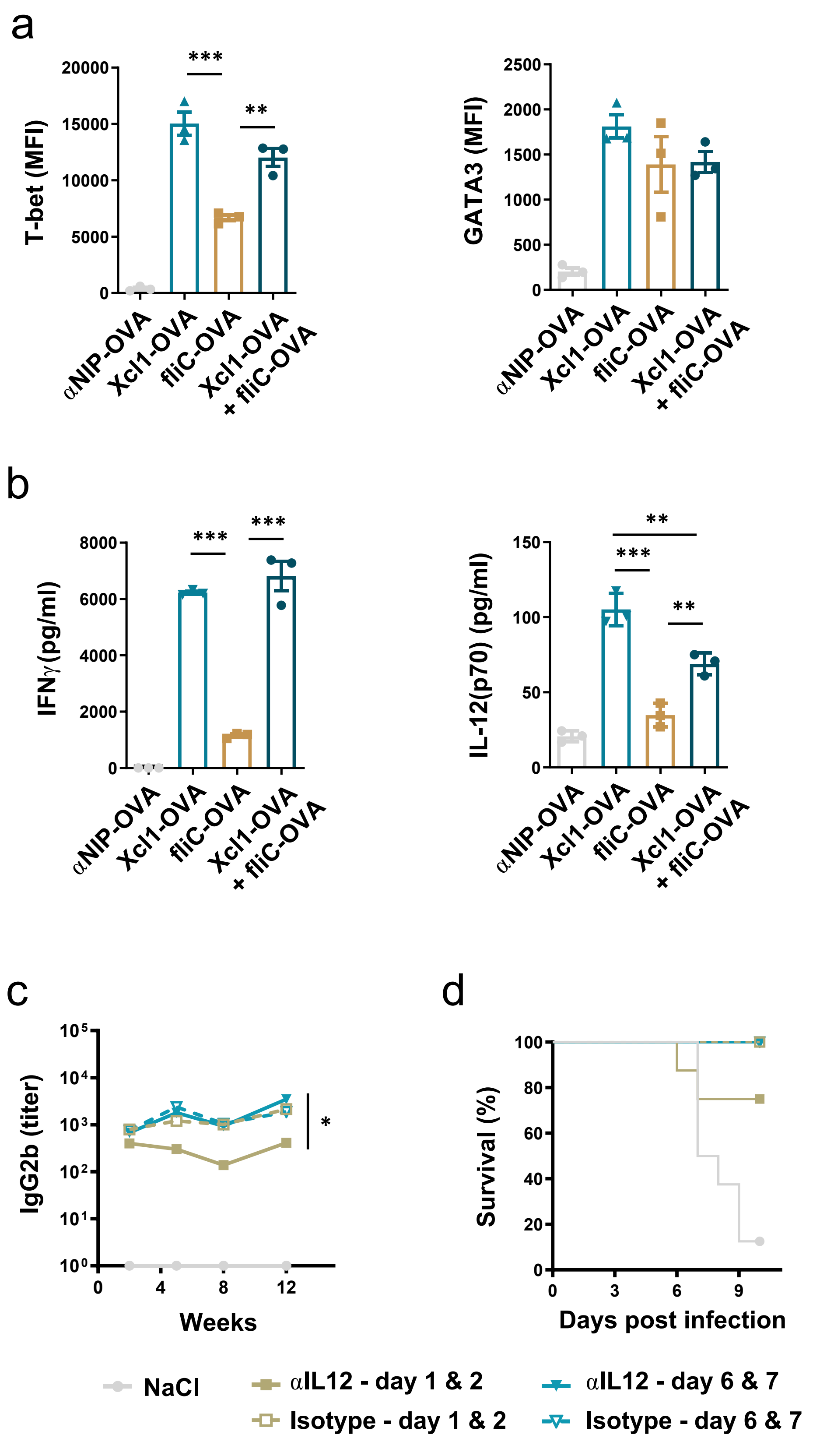

Supplement: Supplementary file 1 [file Presentation_1.pdf]
